# Adaptive leadership curriculum for Indian paramedic trainees

**DOI:** 10.1186/s12245-016-0103-x

**Published:** 2016-02-20

**Authors:** Aditya Mantha, Nathaniel L. Coggins, Aditya Mahadevan, Rebecca N. Strehlow, Matthew C. Strehlow, S.V. Mahadevan

**Affiliations:** Stanford Emergency Department, 300 Pasteur Dr., Alway Bldg M121 MC 5119, Stanford, CA 94305 USA; Stanford Emergency Medicine International, Division of Emergency Medicine, Stanford School of Medicine, Stanford, USA; Department of Medicine, David Geffen School of Medicine, University of California Los Angeles, Los Angeles, USA; University of California San Diego, San Diego, USA; University of California Berkeley, Berkeley, USA; Division of Emergency Medicine, Stanford School of Medicine, Stanford, USA

**Keywords:** Prehospital education, Interpersonal skills, International emergency medicine

## Abstract

**Background:**

Paramedic trainees in developing countries face complex and chaotic clinical environments that demand effective leadership, communication, and teamwork. Providers must rely on non-technical skills (NTS) to manage bystanders and attendees, collaborate with other emergency professionals, and safely and appropriately treat patients. The authors designed a NTS curriculum for paramedic trainees focused on adaptive leadership, teamwork, and communication skills critical to the Indian prehospital environment.

**Methods:**

Forty paramedic trainees in the first academic year of the 2-year Advanced Post-Graduate Degree in Emergency Care (EMT-paramedic equivalent) program at the GVK-Emergency Management and Research Institute campus in Hyderabad, India, participated in the 6-day leadership course. Trainees completed self-assessments and delivered two brief video-recorded presentations before and after completion of the curriculum.

**Results:**

Independent blinded observers scored the pre- and post-intervention presentations delivered by 10 randomly selected paramedic trainees. The third-party judges reported significant improvement in both confidence (25 %, *p* < 0.01) and body language of paramedic trainees (13 %, *p* < 0.04). Self-reported competency surveys indicated significant increases in leadership (2.6 vs. 4.6, *p* < 0.001, *d* = 1.8), public speaking (2.9 vs. 4.6, *p* < 0.001, *d* = 1.4), self-reflection (2.7 vs. 4.6, *p* < 0.001, *d* = 1.6), and self-confidence (3.0 vs. 4.8, *p* < 0.001, *d* = 1.5).

**Conclusions:**

Participants in a 1-week leadership curriculum for prehospital providers demonstrated significant improvement in self-reported NTS commonly required of paramedics in the field. The authors recommend integrating focused NTS development curriculum into Indian paramedic education and further evaluation of the long term impacts of this adaptive leadership training.

## Background

Paramedic trainees in developing countries often face complex and chaotic clinical environments that demand effective leadership, communication, and teamwork. Providers must rely on such non-technical skills (NTS) to manage dozens of bystanders and attendees, collaborate with other emergency professionals, and safely and appropriately treat patients [1–5]. The Accreditation Council for Graduate Medical Education (ACGME) identifies team management as a core competency for graduate emergency medicine trainees [6]. While NTS have been shown to correlate with technical proficiency [7, 8], there is significant variability in terminology, training modalities [5], and evaluation of NTS [2, 3, 9]. English may be the third or fourth language for many, posing an additional barrier to training. As yet, there is no standardized NTS training for India’s nascent paramedic profession. Here, we describe the development and impact of a cross-cultural paramedic NTS curriculum for Indian paramedics.

Until recently, India lacked a centralized system for prehospital emergency medical care analogous to the 9-1-1 system utilized in the USA. In August 2005, the GVK-Emergency Management and Research Institute (GVK-EMRI), a non-profit organization based in the state of Andhra Pradesh, India, launched the first government-sponsored ambulance service under a single call number (1-0-8). The system has since grown to serve 15 Indian states and 2 union territories with a fleet of over 10,000 ambulances [10]. In 2007, GVK-EMRI partnered with the Stanford University School of Medicine to offer India’s first 2-year advanced life support paramedic training program as a means of training, the EMS professionals required to support the new ambulance service.

Our previous work identified several elements of clinical practice that Indian paramedic trainees found challenging (AM, MCS, SVM: "Anthropological analysis of the emergence of the prehospital paramedic in India", unpublished) [ 11]. Crowd control and personal and patient safety were highlighted as primary concerns. The lack of public awareness regarding the role of emergency responders, as well as the differences in culture, religion, and language between patients and emergency responders resulted in paramedics feeling resistance from bystanders on-scene. To address these challenges, we designed an NTS curriculum for Indian paramedic trainees focused on strengthening on-scene leadership, teamwork, and public speaking skills. In July 2013, we conducted this 1-week leadership curriculum at the GVK-EMRI campus in Hyderabad, India, and assessed its impact on the paramedic trainees.

## Methods

### Study population

Forty paramedic trainees in the first academic year of the 2-year Advanced Post-Graduate Degree in Emergency Care (US paramedic equivalent) program at the GVK-EMRI campus in Hyderabad, India, participated in the 6-day leadership curriculum. Of the 40 paramedic trainees, 28 were male and 12 were female. The trainees varied in age from 24 to 48 years and primarily hailed from the Indian states of Gujarat (*n* = 20, median age = 30 years) and Chhattisgarh (*n* = 15, median age = 38 years). Three students who participated in the curriculum were not present for either the pre- or post-intervention survey and were excluded from the analysis.

### Educational intervention

**Fig. 1:**
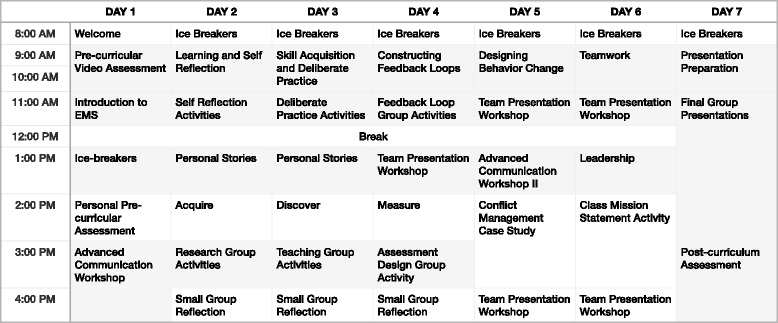
Leadership curriculum schedule

The 6-day leadership curriculum was divided into morning lectures, which reviewed current research on leadership and skill development, and afternoon interactive breakout sessions, which focused on communication and teamwork (Fig. 1). Each day concluded with group discussions on ethics and personal experiences in Indian prehospital care. The curriculum culminated in small group presentations delivered by paramedic trainees to the staff, management, and instructors at EMRI.

The theoretical foundation for the course combines the adaptive leadership models developed by Ronald Heifetz at Harvard University’s Kennedy School of Government [12, 13] and the U.S. Army [14] with team-based safety and emergency management processes crafted by the global aviation industry [2, 8]. Since many situations cannot be resolved by technical mastery alone, adaptive leadership involves analyzing complex situations, identifying available resources and required expertise, formulating a strategy in real-time, and coordinating multiple stakeholders [15]. Our curriculum adapted clinical vignettes and ethical case studies from current events and challenges in Indian healthcare.

### Video assessment

Trainees delivered 2-min video-recorded presentations on randomly selected topics before and after participating in the 6-day leadership curriculum. The order of trainees was also randomized. Each trainee was given 2 min to prepare their topic before presenting to their classmates. Topics were limited to simple, familiar topics: describing family and friends, detailing favorite foods, locations, and sports, and describing the functions of common objects.

To evaluate the efficacy of an objective video-based assessment, we randomly selected video footage of 10 students to process into 30-s clips. For each 2-min pre- and post-intervention, the continuous 30-s interval that contained the most speech was selected. Audio quality was boosted and hue, saturation, and contrast adjusted to create pre- and post-videos of comparable quality for each student. A unique three-digit identifier was embedded within each clip.

Independent judges were recruited from Amazon Mechanical Turk platform to rate pre- and post-intervention videos. The platform allocated 30 native English-speaking judges to rate each of 20 clips using a Likert-style scale (1 = poor, 2 = below average, 3 = average, 4 = above average, 5 = excellent). Judges were provided a nominal compensation of $0.05 per 30-s clip as per the Mechanical Turk platform guidelines. Judges rated and scored the presentations on overall quality, body language (gestures, posture, and eye contact), emotional engagement (enthusiasm, expressions, and emotions), content (clarity, depth, and organization), auditory delivery (pacing, volume, and tone), confidence, and English language competency. Judges were blinded as to whether the video clip was pre- or post-intervention. The study was exempted from institutional review by Stanford University IRB.

### Student self-assessment

**Fig. 2:**
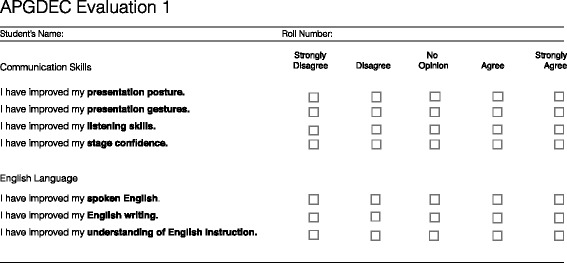
Self-reported evaluation of communication skills questionnaire

**Fig. 3:**
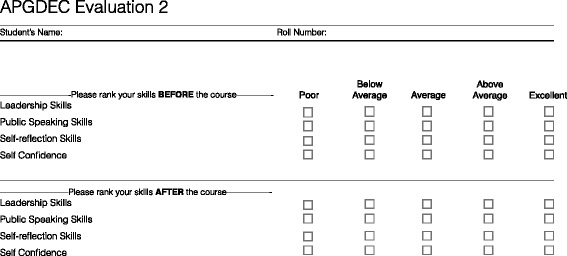
Self-reported evaluation of professional skills questionnaire

Students reported any self-perceived changes in their skills following participation in the 6-day curriculum on two surveys that used Likert-style scales. On the first survey, trainees rated their agreement with statements describing improvements in communication and English language comprehension and literacy (1 = strongly disagree, 2 = disagree, 3 = no opinion, 4 = agree, 5 = strongly agree; Fig. 2). On the second survey, trainees ranked their pre-and post-curriculum competence in leadership, teamwork, public speaking, and confidence (1 = poor, 2 = below average, 3 = average, 4 = above average, 5 = excellent; Fig. 3).

### Statistical analysis

Assessment scores were compared using pair-wise Student’s *t* tests. Analyses were performed in R version 3.0.2 (R Core Team, Vienna, Austria).

## Results

Independent blinded observers scored 10 randomly selected pre- and post-intervention presentations delivered by Indian paramedic trainees (Table 1). While the general trend was improvement across all evaluation categories, the judges reported significant improvement in both confidence (25 %, *p* < 0.01) and body language of paramedic trainees (13 %, *p* < 0.04).

**Table 1:** Pre- versus post-intervention video presentation scores (*n* = 10) of Indian paramedic trainees participating in a 6-day leadership curriculum in July 2013

Presentation skill	Pre-curriculum mean (± s.d.)	Post-curriculum mean (± s.d.)	Difference (%)	*p* value
Overall	2.90 ± 0.58	3.23 ± 0.38	+11	0.09
Body language	3.05 ± 0.67	3.46 ± 0.65	+13	0.04
Emotional engagement	3.14 ± 0.62	3.52 ± 0.66	+12	0.09
Content	2.83 ± 0.49	2.98 ± 0.54	+5	0.42
Auditory	3.11 ± 0.49	3.35 ± 0.37	+8	0.14
Confidence	2.89 ± 0.65	3.60 ± 0.71	+25	0.01
English skills	2.85 ± 0.53	2.76 ± 0.58	+4	0.68

When comparing pre- and post-curriculum self-reported competencies (Table 2), paired samples *t* tests indicated significant increases in leadership (2.6 vs. 4.6, *p* < 0.001, *d* = 1.8), public speaking (2.9 vs. 4.6, *p* < 0.001, *d* = 1.4), self-reflection (2.7 vs. 4.6, *p* < 0.001, *d* = 1.6), and self-confidence (3.0 vs. 4.8, *p* < 0.001, *d* = 1.5).

**Table 2:** Self-perceived non-technical skill aptitude of Indian paramedic trainees (*n* = 37) participating in a 6-day leadership curriculum in July 2013

Self reported improvement	Pre-curriculum mean (± s.d.)	Post-curriculum mean (± s.d.)	Improvement (%)	*p* value
Leadership skills	2.55 ± 0.36	4.59 ± 0.18	80	<0.001
Public speaking skills	2.89 ± 0.37	4.57 ± 0.19	58	<0.001
Self-reflection skills	2.68 ± 0.34	4.57 ± 0.18	71	<0.001
Confidence	3.03 ± 0.38	4.78 ± 0.15	58	<0.001

All participating paramedic trainees (*n* = 37) completed a survey at the conclusion of their leadership training (Table 3). Nearly all trainees perceived improvements in their posture (4.7 ± 0.6; 22 % agree, 76 % strongly agree), presentation gestures (4.5 ± 0.7; 38 % agree, 59 % strongly agree), listening skills (4.7 ± 0.6; 16 % agree, 81 % strongly agree), and stage-confidence (4.8 ± 0.4; 16 % agree, 84 % strongly agree). Trainees overwhelmingly responded positively to statements about their English language development in regards to speech (4.3 ± 0.6; 57 % agree, 41 % strongly agree), writing (4.3 ± 0.7; 54 % agree, 38 % strongly agree), and comprehension (4.3 ± 0.7; 54 % agree, 41 % strongly agree).

**Table 3:** Agreement with statements describe self-perceived improvement among Indian paramedic trainees (*n* = 37) participating in a 6-day leadership curriculum in July 2013

Statement	Strongly agree (%)	Agree (%)	No opinion (%)	Disagree (%)	Strongly disagree (%)
Communication skills					
I have improved my presentation posture	28 (76)	8 (22)	0	1 (3)	0
I have improved my presentation gestures	22 (59)	14 (38)	0	1 (3)	0
I have improved my listening skills	30 (81)	6 (16)	0	1 (3)	0
I have improved my stage confidence	31 (84)	6 (16)	0	0	0
English Language					
I have improved my spoken English	15 (41)	21 (57)	0	1 (3)	0
I have improved my English writing	14 (38)	20 (54)	2 (5)	1 (3)	0
I have improved my understanding of English instruction	15 (41)	20 (54)	0	2 (5)	0

## Discussion

Our novel 6-day paramedic leadership curriculum for Indian paramedic trainees resulted in significant improvement of their communication skills and confidence. Ninety-nine percent of trainees reported improvement in their English language and presentation skills at the conclusion of the course. In a random subset of participants, blinded third-party judges identified improvements in the confidence and body language of paramedic trainees based on the video presentation. Students and clinical instructors provided strong anecdotal evidence for the curricular emphasis on student participation and personal development. For many trainees, the final group presentation was their first public speech in English delivered in front of an audience.

This is the first NTS curriculum developed for Indian prehospital and emergency care providers. Our results demonstrate that NTS, specifically leadership, communication, and teamwork, can be effectively integrated into clinical prehospital training. Studies examining NTS in emergency care range from leadership [8, 16–17], teamwork [18–21], safety [22–25], and communication [1, 6, 26–30]. Our developed curriculum focused on three of the five major prehospital NTS domains outlined by Shields and Flin [2].

The video grading scheme provided a cost-effective method for blinded assessment of NTS. NTS are generally assessed either by expert observation simulated performance/simulated performance [30, 31] or self-report [30]. While self-reported questionnaires are cost-effective and scalable in comparison to simulation, they inherently suffer from recall and reporting biases [32]. In our study, self-rated improvement was significantly higher than the improvement rated by the independent blinded observers, indicating potential social desirability bias. We have previously shown that expert-rated simulation modalities are effective for international prehospital education [33]. However, simulations or direct clinical observation benefit from well-defined, standardized clinical scenarios and are costly to scale [8] and blind. Our web-based video-assessment methodology offers a scalable, robust mechanism for blinded assessments.

Interpersonal NTS skills extend beyond clinical care. Prehospital care is associated with significant burnout, somatic health problems, PTSD, and increased occupational fatality rate [34]. Though paramedics have a lower prevalence of smoking as compared to the general public, they have an increased propensity for sedentary behavior and early retirement [35]. In qualitative studies, paramedics in established emergency care systems identified poor organizational support, emotionally exhausting cases, chronic care, and lack of professional respect as sources of stress and dissatisfaction [36, 37]. Interpersonal skill training may provide a mechanism to raise awareness about these challenges early in paramedic training. Further research may elucidate the effect, if any, of NTS curricula have on supporting provider health promotion and wellness.

**Fig. 4:**
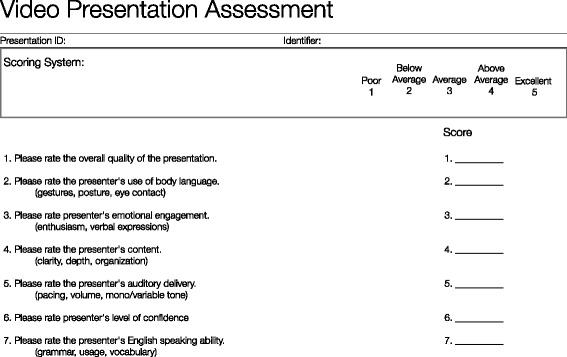
Video-presentation assessment questionnaire

Our study was primarily limited by sample size of the assessment cohort, the duration of the curriculum, the short-term follow-up, and the complex nature of NTS development. Although we had strong anecdotal evidence of improvement, we aimed to pilot a video-based third-party assessment. Anonymous, blinded judges evaluated pre- and post-intervention oral presentations of 10 randomly selected students on a 7-item instrument (Fig. 4). The video assessment was underpowered with only a 40 % chance of detecting a strong effect (*d* = 0.8). However, to reduce rater bias and increase reliability, we had 30 different judges rate each video clip on the 5-item Likert scale. Due to the short duration of the course, our primary outcome measures focused on public speaking because other NTS domains, like leadership and teamwork, take time, experience, and reflection to develop. We plan to utilize the video-based assessment tool in the future to assess multiple NTS, to extend the duration of follow-up, and to assess a larger cohort of paramedic trainees. Finally, to reduce the inherent bias in repeated testing of a skill, we randomized the order and subject of presentation of students.

## Conclusions

Short-term outcomes of this leadership curriculum demonstrate a significant increase in paramedic trainees’ self-perceived confidence, communication, and leadership skills. Blinded third-party observers corroborated the improvement in confidence and body language of a subset of trainees. Our video-based assessment pilot demonstrated the feasibility of cost-effective, blinded evaluation of NTS skills using standardized tasks. We recommend integrating focused NTS development curriculum into Indian paramedic education and further evaluation of the long-term impacts of this adaptive leadership training.

## Abbreviations

GVK-EMRI: GVK-Emergency Management and Research Institute
NTS: non-technical skills
